# Electroporation: A Sustainable and Cell Biology Preserving Cell Labeling Method for Adipogenous Mesenchymal Stem Cells

**DOI:** 10.1089/biores.2019.0001

**Published:** 2019-03-29

**Authors:** Kathrin von der Haar, Rebecca Jonczyk, Antonina Lavrentieva, Birgit Weyand, Peter Vogt, André Jochums, Frank Stahl, Thomas Scheper, Cornelia A. Blume

**Affiliations:** ^1^ Institute of Technical Chemistry, Leibniz University Hannover, Hannover, Germany.; ^2^ Department of Plastic Hand and Reconstructive Surgery, Hannover Medical School Hannover, Hannover, Germany.

**Keywords:** AD-hMSCs, cell labeling, electroporation, stem cell therapy, transfection

## Abstract

Human mesenchymal stem cells derived from adipose tissue (AD-hMSCs) represent a promising source for tissue engineering and are already widely used in cell therapeutic clinical trials. Until today, an efficient and sustainable cell labeling system for cell tracking does not exist. We evaluated transient transfection through electroporation for cell labeling and compared it with lentiviral transduction for AD-hMSCs. In addition, we tested whether nonsense DNA or a reporter gene such as enhanced green fluorescent protein (EGFP) is the more suitable label for AD-hMSCs. Using electroporation, the transfection efficiency reached a maximal level of 44.6 ± 1.1% EGFP-positive cells after selective and expansive cultivation of the mixed MSC population, and was 44.5 ± 1.4% after gene transfer with Cyanin3-marked nonsense-label DNA, which remained stable during 2 weeks of nonselective cultivation (37.2 ± 4.7% positive AD-hMSCs). Electroporation with both nonsense DNA and pEGFP-N1 led to a slight growth retardation of 45.2% and 59.1%, respectively. EGFP-transfected or transduced AD-hMSCs showed a limited adipogenic and osteogenic differentiation capacity, whereas it was almost unaffected in cells electroporated with the nonsense-label DNA. The nonsense DNA was detectable through quantitative real-time polymerase chain reaction for at least 5 weeks/10 passages and in differentiated AD-hMSCs. EGFP-labeled cells were trackable for 24 h *in vitro* and served as testing cells with new materials for dental implants for 7 days. In contrast, lentivirally transduced AD-hMSCs showed an altered natural immune phenotype of the AD-hMSCs with lowered expression of two cell type defining surface markers (CD44 and CD73) and a relevantly decreased cell growth by 71.8% as assessed by the number of colony-forming units. We suggest electroporation with nonsense DNA as an efficient and long-lasting labeling method for AD-hMSCs with the comparably lowest negative impact on the phenotype or the differentiation capacity of the cells, which may, therefore, be suitable for tissue engineering. In contrast, EGFP transfection by electroporation is efficient but may be more suitable for cell tracking within cell therapies without MSC differentiation procedures. Since current protocols of lentiviral gene transduction include the risk of cell biological alterations, electroporation seems advantageous and sustainable enough for hMSC labeling.

## Introduction

Human mesenchymal stem cells (hMSCs) that are present in a high quantity, for example, in human adipose tissue (AD-hMSCs)^1–3^ are frequently used in regenerative medicine,^4^ since these cells can be differentiated in various cell types.^1,5^ Despite being only multipotent, they demonstrate great therapeutic potential through the release of growth factors, chemokines, or antimicrobial signal molecules.^6,7^ A sustainable labeling of hMSCs is relevant to track their way into damaged target tissue and as a forensic test in cases with occurring tumors after cell donation. Substantial manipulations of hMSCs often comprise an increased tendency for carcinogenesis and it may, therefore, be essential to discriminate these donated cells from native cells.^8–10^ The use of inherent native sequences (e.g., karyotyping) for identification is only plausible after nonautologous transplantation, whereas non-native cell characteristics must be used after autologous transplantation. Therefore, we tested unique synthetic fluorescent nonsense DNA^11^ in comparison with a reporter gene (enhanced green fluorescent protein or EGFP) as cell marker. EGFP can be detected, for example, by *in vivo* flow cytometry at available body regions.^12^ The efficiency of transfecting primary cells and especially stem cells is usually not as high as in cell lines^13–15^ and some transfection techniques for AD-hMSCs are questioned to affect cell biology in terms of proliferation or differentiation, affecting the therapeutic use.^16^ In general, only stable transfection methods with genomic integration of target DNA are suggested to be sustainable enough for cell therapy, whereas after transient transfection, target DNA diminishes by dilutional effects during cell division.^11,17^ On the contrary, viral presence—after stable DNA transfer—may produce immunogenicity, cytopathic effects, cancerogenicity, or severe toxicity in the recipient,^18–21^ and this technique, therefore, requires a large number of safety measures as a prerequisite for its performance.^22^

Therefore, it was the aim of our study to develop a transient transfection protocol for AD-hMSCs with high efficiency. Protocols comprising cationic lipids, polymers (e.g., polyethylenimine),^22–24^ or chemical transfection based on CaCl_2_/DNA precipitation^22^ bear the risk of cytotoxicity^22^ and have not proven to be very efficient in AD-hMSCs.^25–27^ Physical methods are reported with high transfection efficiency. Among the different complicated and expensive physical methods such as magnet-mediated transfection, biolistic particle delivery, or microinjection,^28–33^ we decided for electroporation that is relatively easy and cheap. Here an electrical field is applied to permeabilize the cells for DNA transfer.^22,28^ Our protocol should aim for number of cells high enough for clinical applications and sustainable enough to be applied for cell tracking over a long time but with the least possible impact on cell biology.

## Materials and Methods

### Cell cultivation

Primary AD-hMSCs^29^ were isolated and identified by immune phenotype and functional characteristics as defined by the International Society for Cellular Therapy^5^ comprising the presence of CD105, CD73, and CD90, and the absence of CD45, CD34, CD14 or CD11b, CD79α or CD19, and human leukocyte antigen DR isotype (HLA-DR) surface molecules. Cells in passage 2 were cultivated at 37°C in complete medium (minimum essential medium eagle alpha medium; Gibco, Germany), 10% human serum AB (c.c.pro GmbH, Germany), 0.5% gentamycin (Biochrom, Germany) in a T175 culture flask (Sarstedt, Germany) in humidified atmosphere (5% CO_2_/21% O_2_). At 80% confluency, AD-hMSCs were harvested through Accutase©-treatment, counted, and DNA transfer was performed.

### Transfection methods

For electroporation, detached AD-hMSCs were resuspended in hypo-osmolar electroporation buffer (Eppendorf, Germany). According to the literature,^27,30,31^ 10^6^ cells and 20 μg linearized plasmid pEGFP-N1 (4.7 kb; EGFP production under control of the cytomegalovirus (CMV) promotor; cat. no. 6085-1; ClonTech Laboratories, Inc., USA) were transferred into a 4 mm gap electroporation cuvette (BioRad, Germany) and electroporated using an X-cell pulser (BioRad) and a square-wave pulse (50–200 μs) of 400–700 V, and DNA concentrations of 5–25 μg. Electroporated cells were analyzed on days 3, 17, and 31 after the transfer. Selection was performed between days 3 and 17 using complete medium with 200 μg/mL G418 bisulfate.

In addition, 6.8 nmol small synthetic fluorescent-labeled nonsense DNA (Integrated DNA Technologies, Inc., USA; labeled with Cyanine3; Supplementary Table S1) was transferred into AD-hMSCs through electroporation before cell analysis 3 and 14 days later.

To compare with a stable DNA transfer protocol, we performed lentiviral transduction using a second-generation lentiviral vector system encoding for EGFP (pLVTHm, 11 kb; EGFP production under control of the EF1α promoter) as described before.^32^ Cells were suspended in complete medium supplemented with 50 μg/mL protamine sulfate.

### Flow cytometry analysis

The number of marker-positive transfected cells was determined using flow cytometry (Epics XL-MCL; Beckman Coulter, USA) at the time points indicated. Data were analyzed using WinMDI 2.9 (Joe Trotter, Purdue University, USA). *egfp* expressing lentiviral-transduced cells were isolated for analysis through fluorescence-activated cell sorting (MoFlo XDP; Beckman Coulter).

### Quantitative real-time polymerase chain reaction analysis of transfected cells

For quantitative real-time polymerase chain reaction (qRT-PCR) analysis, total DNA was isolated using DNeasy Blood and Tissue Kit, messenger RNA was gained utilizing RNeasy Mini Kit (each from Qiagen, Germany), and transcribed into complementary DNA as described previously.^33^ qRT-PCR was performed using IQTM5 real-time PCR detection system, 12.5 μL IQTMSYBR^®^ Green Supermix (both BioRad), 1 μL primermix (0.25mM; see Supplementary Table S2), and 10 ng DNA. Absolute DNA concentrations were determined using sample threshold cycle numbers and a calibration curve (Supplementary Figs. S1 and S2).^34^

### Cell growth

To determine colony-forming units (CFUs), cells were plated in 100 mm cell culture dishes at a concentration of 50 cells/cm^2^, cultivated for 8 days, fixed with 4% paraformaldehyde in phosphate buffered saline (PBS) for 20 min at room temperature (RT) and stained with crystal violet (0.5% in 70% methanol). CFUs were counted using an Olympus IX50 microscope (through Olympus CMOS camera SC30, Germany).

### Senescence

Senescence was analyzed using a β-Galactosidase Cell Staining Kit (Cell Signaling Technology, Inc., USA) according to the manufacturer's instructions. In addition to negative control cells, senescence was induced in untreated AD-hMSCs by cultivation in high-glucose medium (4.5 g/L) for 21 days.^35^

### MSC phenotype analysis

Untreated and transfected cells were analyzed regarding their immunophenotype, including CD44, CD73, CD90, and CD105, and negative CD31, CD34, and CD45^5^ using either phycoerythrine (PE)-(CD44, CD45, and CD73); fluorescein isothiocyanate (FITC)-(CD31, CD34, and CD90); or PE-CF594-(CD105)-conjugated antibodies. As negative control, cells were stained with PE, PE-Cy-5, FITC, or PE-CF594-conjugated mouse IgG1, or PE-conjugated mouse IgG2b isotype control antibodies (all BD Bioscience, Germany). Immune-stained cells were analyzed using the Epics XL-MCL flow cytometer. Generated data were analyzed using WinMDI 2.9. At least 10^4^ viable cells were gated in a dot plot of forward versus side scatter.

### Differentiation capacity

To determine differentiation capacity, cells were seeded in six-well plates (3000 cells/cm^2^) and cultivated in complete medium until reaching confluency. Cells were cultivated in osteogenic or adipogenic differentiation medium (Miltenyi Biotec, Germany) for 21 days and fixed with 4% paraformaldehyde. Tissue-specific staining was performed as described hereunder, differentiated cells were compared with transfected but nondifferentiated (negative control) and untreated differentiated AD-hMSCs (positive control). Adipogenic-treated cells were washed with 60% isopropanol at RT before addition of Oil Red O (2 g/L in 60% isopropanol). Osteogenic-treated cells were incubated with Alizarin Red (20 g/L in double distilled water) for 45 min at RT in the dark. For counterstaining, 4’,6-diamidino-2-phenylindole dihydrochloride (Sigma Aldrich Chemie GmbH, Germany) staining was performed in the dark for 15 min at RT. Staining was analyzed using phase contrast or fluorescence microscopy (U-MWU filter set with BP330-385 [exciter filter], BA420 [barrier filter]).

Adipogenic differentiation was semiquantitatively analyzed through the red pixel count in representative images using Adobe Photoshop 6.0. The grade of osteogenic differentiation was quantified after extraction of the Alizarin Red dye using hexa-decyl-pyridinium-chloride-monohydrate (Sigma Aldrich Chemie GmbH; 10% [w/v] in PBS) and by photometry at a wavelength of 550 nm (Epoch; BioTek Instruments, Inc., USA; calibration curve: 0.13–4.28 M).

### *In vitro* tracking of EGFP-labeled AD-hMSCs

AD-hMSCs electroporated with pEGFP-N1 were imaged for 24 h every 2 min using the incubator microscope LumaScope 600 (Etaluma, Inc., USA) at a magnitude of 40 × (LUCPlanFLN40X, Olympus, Japan) and using green fluorescence (473–491 nm excitation and 502–561 nm emission Semrock Brightline^®^ Pinkel). Single cell tracking was performed using Fijis ImageJ and the Plugin Track Mate.^36^

### EGFP-positive AD-hMSCs imaged on sand-blustered titan plates

EGFP-positive AD-hMSCs (2000 cells/cm^2^) were cultivated on titan plates in 24-well plates for 7 days under static or dynamic conditions with a medium flow of 0.1 mL/min in a specially developed bioreactor. Cells were washed with PBS and fixed with 4% paraformaldehyde. Phalloidin staining visualized actin filaments (0.72 μL phalloidin-iFluor 555 Reagent in 720 μL 1% BSA in PBS; Abcam, Great Britain). Optical analysis was performed at a magnitude of 20 × using fluorescence microscopy (EGFP detection: excitation: 460–495 nm, emission: 510 nm; phalloidin-detection: excitation: 530–550 nm, emission: 565 nm).

### Statistical analysis

Results are given as mean values ± standard deviation with *n* = 3 experiments, measured in triplicates. For qRT-PCR analysis, three independent cultures were pooled to guarantee successful DNA/RNA isolation; combined samples were measured in triplicates. *F*-tests were performed before two-sided *t*-tests were performed with levels of significance as indicated.

## Results

### Transfection efficiencies

**FIG. 1.**
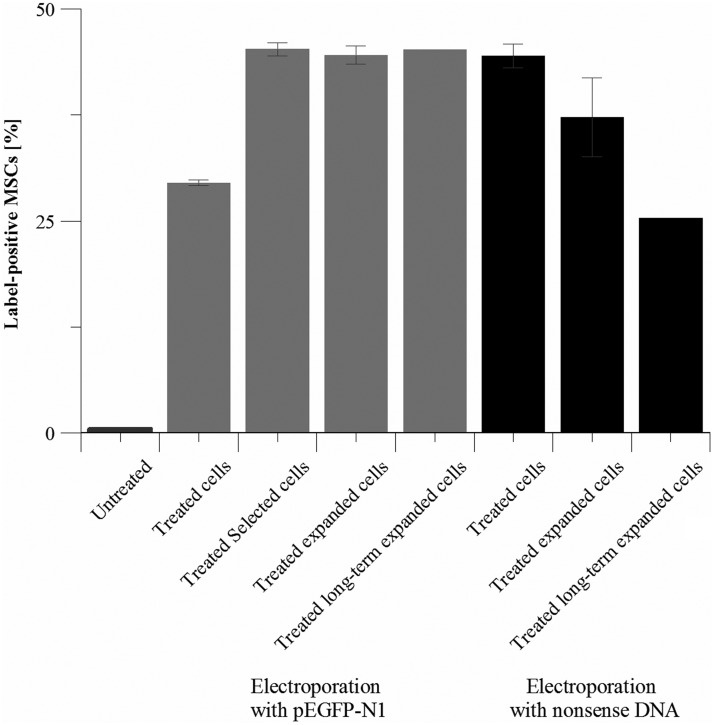
Transfection efficiencies of all performed electroporation experiments with AD-hMSCs. Displayed are percentages of label-positive cells before electroporation (untreated cells), after electroporation with pEGFP-N1 or nonsense DNA, after selection (treated selected cells), after additional 14 days of cultivation (treated expanded cells), and after long-term cultivation (treated long-term expanded cells). Electroporation with pEGFP-N1 led to *egfp* expression; nonsense DNA contained Cy3 dye. Number of cells containing these fluorescent markers was obtained using flow cytometry. *n* = 3, mean ± SD. AD-hMSCs, human mesenchymal stem cells derived from adipose tissue; EGFP, enhanced green fluorescent protein; SD, standard deviation.

Electroporation of 10^6^ AD-hMSCs with pEGFP-N1 reached a transfection efficiency of 29.5 ± 0.4% cells, which increased to 45.3 ± 0.8% EGFP-positive cells after selection with G418 bisulfate. This rate stayed stable at 44.6 ± 1.1% (Fig. 1, gray) for the next 14 days and was in accordance with the transfection efficiency after gene transfer of nonsense DNA (44.5 ± 1.4%), which slightly decreased after another 14 days of nonselective cultivation (37.2 ± 4.7%; Fig. 1, dark gray).

### Sustainability of target DNA after transfection

To test marker sustainability in a long-term cultivation, electroporated AD-hMSCs after day 3 (nonsense DNA) or day 17 (EGFP, days 3–17: selection) were cultivated for additional 32 days (after 28 days, cells were cryoconserved and subsequently cultivated for another 4 days). Thereafter, 45.2% cells were EGFP positive (Fig. 1, gray) or 25.3% of all cells were nonsense DNA-positive (Fig. 1, dark gray), respectively.

Nonsense DNA and *egfp* DNA were detectable in high amounts after electroporation and further cultivation of 14 days as well as after long-term cultivation. After 21 days of differentiation into adipocytes, which started 3 days after electroporation, *egfp* DNA decreased, whereas nonsense DNA was still present in large amounts (Supplementary Fig. S1).

### EGFP expression

**FIG. 2.**
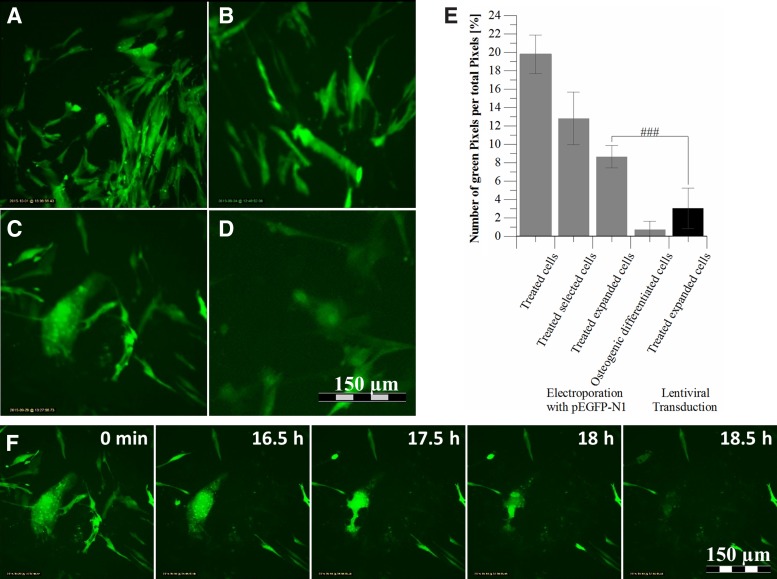
*egfp* expression of electroporated or lentiviral-transduced AD-hMSCs. Exemplary images of AD-hMSCs electroporated with pEGFP-N1 are shown: 3 days after electroporation **(A)**, during selection by cultivation in complete medium with 200 μg/mL G418 bisulfate **(B)**, during osteogenic differentiation after selection and 14 days cell expansion **(C)**, after lentiviral transduction **(D)**, comparison of green pixels in **(A–D)** as semiquantitative analysis for differentiation capacity **(E)**. **(F)** Exemplary images during different time points of osteogenic differentiation after selection. All pictures were taken using the incubator microscope LumaScope 600 (Etaluma, Inc., USA) and a 40 × objective (LUCPlanFLN40X; Olympus, Japan). A full time lapse video is shown in Supplementary Movie S3. ^# ^Shows *t*-test comparison of two samples: ^###^*p* ≤ 0.001.

EGFP expression slightly declined during expansion after electroporation and selection, but the fluorescent signal was always significantly stronger in electroporated cells than in the lentivirally transduced cells (Fig. 2A–E, Supplementary Movies S1 and S2).

### Growth retardation

**FIG. 3.**
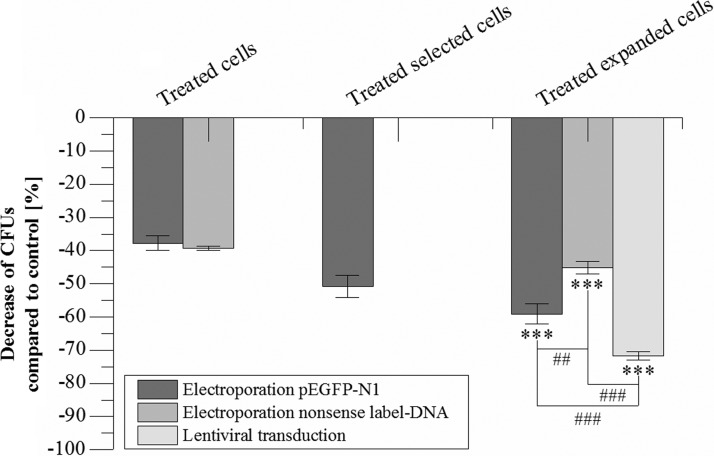
Effects of the transfection/transduction procedure on CFU. Displayed is the decrease of CFUs formed after 8 days of cultivation in complete medium compared with untreated negative control cells ( = 0%). (*n* = 3, mean ± SD; *Shows *t*-test comparison of each sample with the negative control and between two samples: ***p ≤ 0.001; ^#^ shows *t*-test comparison of two samples: ^##^*p* ≤ 0.01; ^###^*p* ≤ 0.001). CFUs, colony-forming units.

CFU formation in comparison with untreated AD-hMSCs was observed after each method of DNA transfer. Figure 3 shows that CFUs were reduced by 37.8% after electroporation in AD-hMSCs with pEGFP-N1 declining further to a loss of 50.8% after selection and to a level of 59.1% after another 14 days of expansion. Fourteen days after electroporation with nonsense DNA, AD-hMSCs showed a growth reduction of 45.2%. In contrast, CFUs were significantly retarded in cells 14 days after lentiviral transduction with plVThM by 71.8%.

### Senescence

None of the tested DNA transfer methods induced senescence in AD-hMSCs (Supplementary Fig. S3).

### MSC immunophenotype

Although the MSC-defining immunophenotype was unaltered after electroporation, the expression of two antigens was reduced after lentiviral transduction (CD44: 77.73%; CD73: 43.04%; Table 1; Supplementary Fig. S4).

**Table 1.** Comparison of Surface Antigen Expression Before (Untreated Cells) and 3 Days After Electroporation (Treated Cells) with pEGFP-N1 or Nonsense Label DNA, After Selection (Treated Selected Cells) and After 14 Days of Cultivation (Treated Expanded Cells), as Well as After Lentiviral Transduction and Subsequent Cultivation (Treated Expanded Cells)

	Untreated cells	Electroporation pEGFP-N1			Electroporation nonsense label DNA		Lentiviral transduction
		Treated cells	Treated selected cells	Treated expanded cells	Treated cells	Treated expanded cells	Treated expanded cells
CD44	99.75%	99.99%	100.00%	99.87%	98.42%	97.68%	77.73%
CD73	99.86%	85.40%	87.34%	88.98%	99.66%	98.74%	43.04%
CD90	99.85%	98.00%	98.32%	92.56%	99.75%	98.14%	98.44%
CD105	99.14%	98.73%	98.85%	98.43%	97.32%	97.67%	100.00%
CD31	0.06%	0.50%	1.67%	0.70%	2.56%	0.13%	0.93%
CD34	0.49%	0.21%	0.24%	1.50%	2.03%	0.51%	0.79%
CD45	0.49%	0.25%	0.69%	1.10%	0.34%	4.05%	0.58%

EGFP, enhanced green fluorescent protein.

### Adipogenic and osteogenic differentiation capacity

Electroporated cells in this study represented a mixed culture of marker-positive and marker-negative AD-hMSCs. Owing to cell agglomeration after differentiation, flow cytometry analysis of differentiated cells was not possible to identify if marker-positive or marker-negative cells were differentiated.

**FIG. 4.**
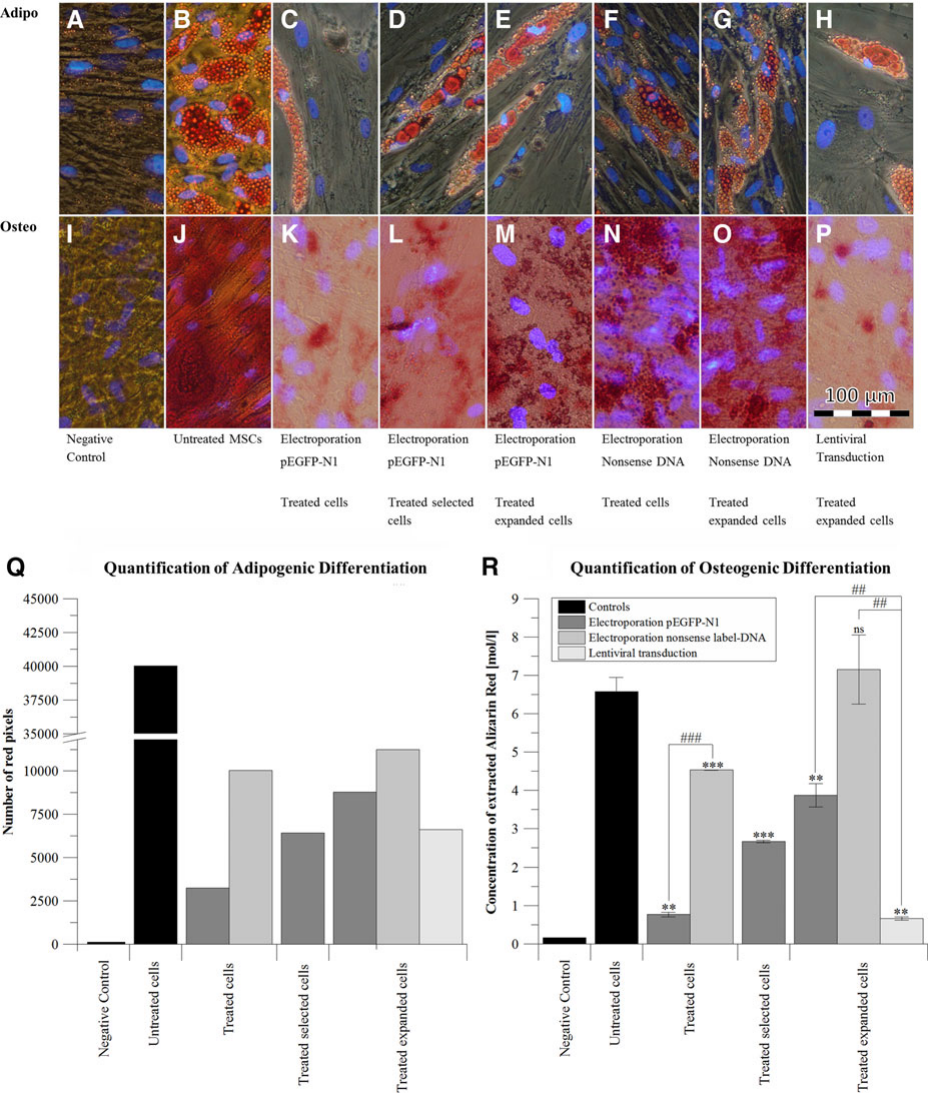
Examination of effects of electroporation and lentiviral transduction on the differentiation potential of AD-hMSCs. Representative sections of staining images in differentiated AD-hMSCs for 23 days are shown exemplarily **(A–P)**. Nuclei were visualized by DAPI counter staining (blue signals). AD-hMSCs were cultivated in adipogenic differentiation medium **(B–H** vs. undifferentiated control cells**)**, **(A)**; accumulated lipid droplets were visualized with Oil Red O **(**first row, red dye, **A–H)** and in osteogenic differentiation medium **(J-P** vs. undifferentiated cells, **I)**; here extracellular calcium accumulation was visualized with Alizarin Red **(**second row, red dye, **I–P)**; **(Q)** a semiquantitative analysis of the number of red pixels in all prepared Oil Red O-stained images was performed to estimate the grade of adipogenic differentiation **(A–H)** using Adobe Photoshop and the function “Color Range” for red colors. **(R)** All osteogenic samples **(I–P)** underwent a dye elution: Alizarin Red was quantified to estimate the grade of osteogenic differentiation after measurements at 550 nm and comparison with a calibration curve (*shows *t*-test comparison of each sample to the positive control: ***p ≤ 0.001; **p ≤ 0.01; ns, *p* > 0.05; ^#^shows *t*-test comparison of two samples: ^##^*p* ≤ 0.01; ^###^*p* ≤ 0.001). DAPI, 4’,6-diamidino-2-phenylindole dihydrochloride; ns, not significantly different.

We image-analyzed adipogenic differentiation according to the number of red-stained lipid droplets in pictures of all differentiated samples (Fig. 4A–H) and by comparison with untreated cells (positive control, Fig. 4B,Q). The grade of adipogenic differentiation was higher in electroporated AD-hMSCs than in lentivirally transduced cells (nonsense DNA electroporation vs. lentiviral transduction: 1.7 times higher; pEGFP-N1 electroporation vs. lentiviral transduction: 1.3 times higher; Fig. 4Q) and the overall highest in nonsense DNA-labeled AD-hMSCs. Adipogenic differentiation proceeded during cell expansion after electroporation (Fig. 4Q, dark gray).

After osteogenic differentiation, calcium accumulation was observed as an indicator in all samples (Fig. 4J–P). It was lowest after lentiviral transduction (Fig. 4P) without recovery throughout cell expansion. After electroporation, calcium concentrations were lower than in untreated cells but increased during further cell expansion. We additionally used Alizarin Red dye—extraction (Fig. 4R) for quantification of osteogenic differentiation compared with the 100% positive control (untreated osteogenic differentiated AD-hMSCs; 6.5 M Alizarin Red). Here calcification was first reduced to 11.7% (0.77 M Alizarin Red) directly after electroporation with pEGFP-N1 but recovered to 40.6% (2.67 M Alizarin Red) after selection and to 59.0% (3.87 M Alizarin Red) after additional cell expansion for 14 days (Fig. 4R, dark gray). In lentivirally transduced AD-hMSCs, calcification was reduced to 10.0% (0.66 M Alizarin Red) even after cell expansion (Fig. 4R, light gray). In AD-hMSCs electroporated with nonsense DNA, calcification was only reduced to 68.95% (4.53 M Alizarin Red), but fully recovered after additional 14 days of cell expansion (7.15 M Alizarin Red; 108.81%) to a level comparable with the positive control.

### *In vitro* tracking of electroporated EGFP-labeled AD-hMSCs

**FIG. 5.**
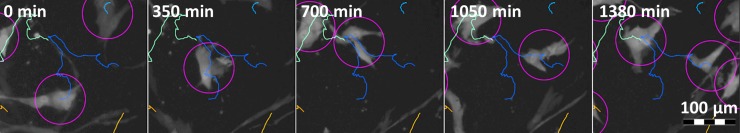
*In vitro* tracking of AD-hMSCs during expansion in complete medium. AD-hMSCs (electroporated with pEGFP-N1 and selected) are shown during expansion in complete medium. Images were recorded using the incubator microscope LumaScope 600 (Etaluma, Inc.) and a 40× objective (LUCPlanFLN40X; Olympus); single cell tracking was performed using Fijis ImageJ and the Plugin TrackMate^42^ with the following settings: LoG detector with blob size of 200 px; LAP tracker with 25 px distance, closing gap distance of 25 px, and a maximum frame gap of 2. Purple circles indicate detected single cells, colored lines indicate cell traces. Here, the focus lies on the single cell with the blue trace line.

EGFP-labeled AD-hMSCs were tracked *in vitro* for 1 day using an incubator microscope, and Fijis ImageJ and the Plugin TrackMate^36^ (exemplary images, Fig. 5). Single cell migration (purple circle, trace depicted in blue) was documented during the first 20 h of differentiation into osteoblasts in EGFP-positive cells (Fig. 2F). Comparatively, cell tracking was performed with nonsense DNA-positive cells (not shown).

### EGFP-positive AD-hMSCs imaged on sand-blustered titan plates

**FIG. 6.**
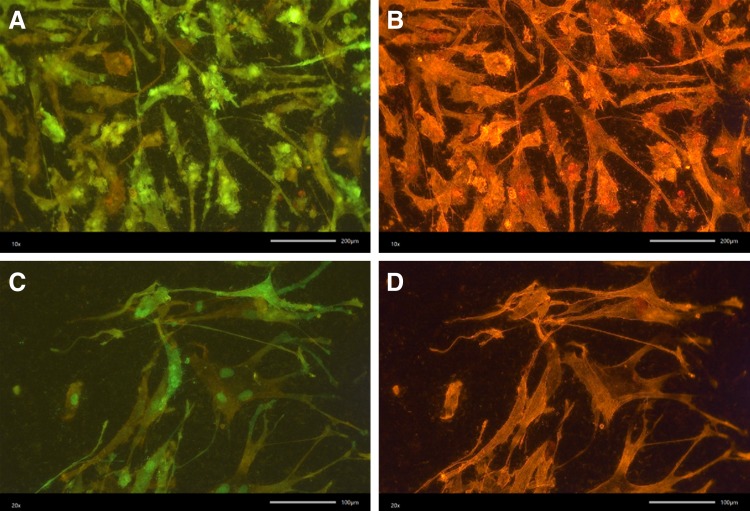
EGFP-positive AD-hMSCs on sand-blustered titan plates used as surface for dental implants. GFP-labeled AD-hMSCs under static conditions **(A, B)** or exposed to laminar flow in a suitable bioreactor with a flow rate of 0.1 mL/min **(C, D)**. EGFP was detected using an excitation wavelength of 460–495 nm and an emission wavelength of 510 nm **(A, C)**, phalloidin-stained actin filaments are shown at an excitation of 530–550 nm and an emission of 575 nm **(B, D)** using fluorescence microscopy, magnitude 20×.

EGFP-positive AD-hMSCs were used as a tool for cell adherence cultivated for 7 days on nontransparent implant materials, which could be analyzed with a standard microscope. This cell test further allowed simultaneous phalloidin staining of actin filaments. The DNA transfer had no apparent influence on cell behavior in this approach (Fig. 6).

## Discussion

Human AD-hMSCs used in several clinical trials bear promising results and are suggested for tissue engineering.^4,37^ For these applications, AD-hMSCs are expanded and substantially manipulated *ex vivo* (relevant for the approval as artificial medicinal product; advanced therapy medicinal product).^37–40^ With a view to identify such cells after transfer to a patient, we developed a reliable protocol for cell labeling. This electroporation protocol is efficient reaching 45% positive cells. It is nondeteriorating for the cells, but stable over the entire injected AD-hMSCs lifespan,^16^ and did not affect cell biology. This nonviral gene delivery method does not pose safety concerns as viral methods do (e.g., mutagenesis, cytotoxicity, and possible immunological reactions).

The transfection efficiency of our protocol was comparable with some previous studies with primary cells^16,28^ and eminently higher than that of studies with different kinds of stem cells and other transient transfection methods (Table 2).^24,25,27,41–43^ According to flow cytometry analysis, our protocol meets clinically relevant number of cell suitable for tissue engineering and cell transplantation.

**Table 2.** Transfection Efficiency of Different Transient Transfection Methods and Different Stem Cell Types in Current Research

Transfection method	Stem cell type	Cell origin organism	Marker	DNA amount	Starting number of cells	Transfection efficiency	Transfection efficiency	Ref.
Electroporation	BMSCs	Rat	β-Gal	8 μg pDRIVE2-CMV	5 × 10^5^	29 ± 3%	β-Gal production decreased by ∼80% (7 days)	50
Nucleofection	BMSCs	Human	EGFP	5 μg pCMV-EGFP-N1	5 × 10^5^	68.2 ± 4.1%	—	49
	BMSCs	Human	hBMP-2	5 μg pCMV-CDNA3-hBMP2	5 × 10^5^	—	Gene expression decreased by ∼99% (14 days)	
Electroporation	BMSCs	Human	EGFP	10 μg pEGFP-N1	10^6^	12%	—	37
Electroporation	ESCs	Human	EGFP	10–60 μg pCAG-EGFP	10^5–^10^6^	≤17.6%	—	19
Lipofection	ESCs	Human	EGFP	10–60 μg pCAG-EGFP	10^5–^10^6^	≤1.5%	—	
Lipofection	BMSCs	Human	EGFP	1 μg pVAX-GFP	1900 (72 h	50 ± 2.8%	Gene expression decreased to ≤10% (10 days)	21
					5700 (72 h	<50%		
	adMSCs	Human	EGFP	1 μg pVAX-GFP	1900 (72 h	33 ± 4.7%	Gene expression decreased to ≤10% (10 days)	
					5700 (72 h	<30%		
	ucMSCs	Human	EGFP	1 μg pVAX-GFP	1900 (72 h	<45%	Gene expression decreased to ≤10% (10 days)	
					5700 (72 h	58 ± 7.1%		
Cationic polymer: PEI	adMSCs	Human	EGFP	1 μg pEGFP-C2	1.5 × 10^5^ (24 h	≤7.11%	—	48
Cationic polymer: PAMAM dendrimers	adMSCs	Human	EGFP	1 μg pEGFP-C2	1.8 × 105 (24 h	≤8.61%	—	20

β-Gal, β-galactosidase; adMSCs, human mesenchymal stem cells derived from adipose tissue; BMSCs, bone marrow stem cells; EGFP, enhanced green fluorescent protein; ESCs, embryonic stem cells; hBMP-2, human bone morphogenetic protein; PAMAM, polyamidoamine; PEI, polyethylenimine; ucMSCs, umbilical cord tissue-derived mesenchymal stem cells.

Although electroporation is a transient gene transfer protocol, cell labeling was sustainable for a total of 35 days (nonsense DNA) or 49 days (EGFP, including 14 days of selection; Fig. 1). This was in contrast to literature reports with radically reduced gene expression in long-time observation of cells after different transfection methods (Table 2).^25,42,43^ EGFP expression was higher after electroporation than after lentiviral transduction. We judge the time intervals for EGFP expression as suitable for cell tracking, since the reported AD-hMSC lifespan after transplantation ranges between 24 h and 4 weeks,^44–50^ but *in vivo* studies still have to be performed.

Nonsense DNA was also present for as long as 35 days (25.3% label-positive cells) with a copy number in target cells that was even higher than that in EGFP-positive cells. This may be due to the smaller plasmid size (Supplementary Fig. 4).^51^

Of note, our electroporation protocol had only a slight negative impact on growth according to CFU formation, and the count of CFUs was higher after electroporation than after lentiviral transduction. Nonsense DNA transfer had a lower growth retarding effect than *egfp* gene transfer. In general, senescence was not detectable in any of the protocols (Supplementary Fig. 3). Electroporation with the reporter gene system used did not affect the cell characteristic immunophenotype of AD-hMSCs, but this was the case after lentiviral transduction. In this study, the expression of two MSC defining surface markers CD73 (43.04%) and CD44 (77.73%) relevant for MSC cell migration^52,53^ was decreased. CD73 plays a role for immune adaption and a CD73 deficiency was repeatedly shown to lead to autoimmune inflammation (both *in vivo* and *in vitro*).^54–56^ We, therefore, suggest lentiviral transduction, at least under conditions described here, to induce unwarranted alterations of MSC cell biology.^5,56^

Electroporation with nonsense DNA, subsequently cultivated for cell recovery, showed an unchanged differentiation capacity for osteogenic differentiation and a comparatively only slightly reduced adipogenic differentiation capacity (Fig. 4). In contrast, EGFP production negatively impacted the adipogenic differentiation and to a smaller extent also the osteogenic differentiation. We suggest this to be due to an overcharge caused by the simultaneous expression of *egfp* and proteins relevant for differentiation. Furthermore, we found a relevant number of dead cells within the population of EGFP-positive cells in our time lapse analysis (Fig. 2F). Of note, disturbance of the differentiation process was much more pronounced after lentiviral transduction.

We could demonstrate that online *in vitro* cell tracking for 24 h and cell identification of AD-hMSCs on nontransparent materials for 7 days were possible.

In conclusion, we suggest electroporation as a highly efficient and cell biology preserving method for DNA delivery in AD-hMSCs. The label-DNA introduced via electroporation was detectable for up to 35 days after electroporation (nonsense DNA) or 49 days (EGFP, including 14 days selection). Our *in vitro* experiments show promising results for future applications of electroporated AD-hMSCs after autologous MSC transplantation, although *in vivo* experiments, for example, using *in vivo* flow cytometry still have to be performed.^57^ Whereas EGFP-labeled AD-hMSCs in tissue engineering are of limited use, since *egfp* expression might hinder cell differentiation, cell labeling using nonsense DNA seems promising since it had only a slight negative impact on cell biology of AD-hMSCs. This transient gene transfer method might, therefore, be the method of choice and is in compliance with ethical standards for therapeutic applications of AD-hMSCs in patients. We envision a forensic test, for example, for discrimination of the genealogy of possibly occurring tumor cells after stem cell transplantation or in tissue engineered products based upon this method.^1–3^

## Supplementary Material

Supplemental data

Supplemental data

Supplemental data

Supplemental data

Supplemental data

Supplemental data

Supplemental data

Supplemental data

Supplemental data

## Declarations

### Availability of data and material

All data generated and analyzed during this study were included in this article and its additional files. The data sets used and/or analyzed during this study are available from the corresponding author on reasonable request.

## Abbreviations Used

β-Gal: β-galactosidase
AD-hMSCs: human mesenchymal stem cells derived from adipose tissue
ATMP: advanced therapy medicinal products
BMSCs: bone marrow stem cells
CD: cluster of determination
CFUs: colony-forming units
DAPI: 4’,6-diamidino-2-phenylindole dihydrochloride
EGFP: enhanced green fluorescent protein
*egfp*: gene encoding for enhanced green fluorescent protein
ESCs: embryonic stem cells
FITC: fluorescein isothiocyanate
hBMP-2: human bone morphogenetic protein
hMSC: human mesenchymal stem cell
MSC: mesenchymal stem cell
ns: not significantly different
PAMAM: polyamidoamine
PBS: phosphate buffered saline
PE: phycoerythrine
PEI: polyethylenimine
qRT-PCR: quantitative real-time polymerase chain reaction
RT: room temperature
SD: standard deviation
ucMSCs: umbilical cord tissue-derived mesenchymal stem cells
