# Supplementary material for: Electroporation: A Sustainable and Cell Biology Preserving Cell Labeling Method for Adipogenous Mesenchymal Stem Cells
Source: Biores Open Access. 2019 Mar 29;8(1):32–44. doi: 10.1089/biores.2019.0001 (PMC6445215; doi:10.1089/biores.2019.0001)
Supplement: Supplemental data [file Supp_Table1.pdf]

Supplementary Data

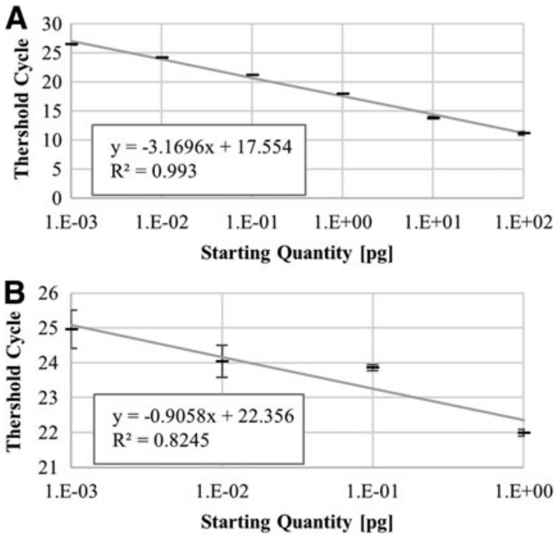

SUPPLEMENTARY FIG. S1. Calibration curves of qRT-PCR analysis. Samples of different known starting quantities of pEGFP-N1 **(A)** or nonsense DNA **(B)** were analyzed by qRT-PCR to obtain calibration curves. Data were exported using BioRadIQ5™ (BioRad, Germany). qRT-PCR, quantitative real-time polymerase chain reaction.

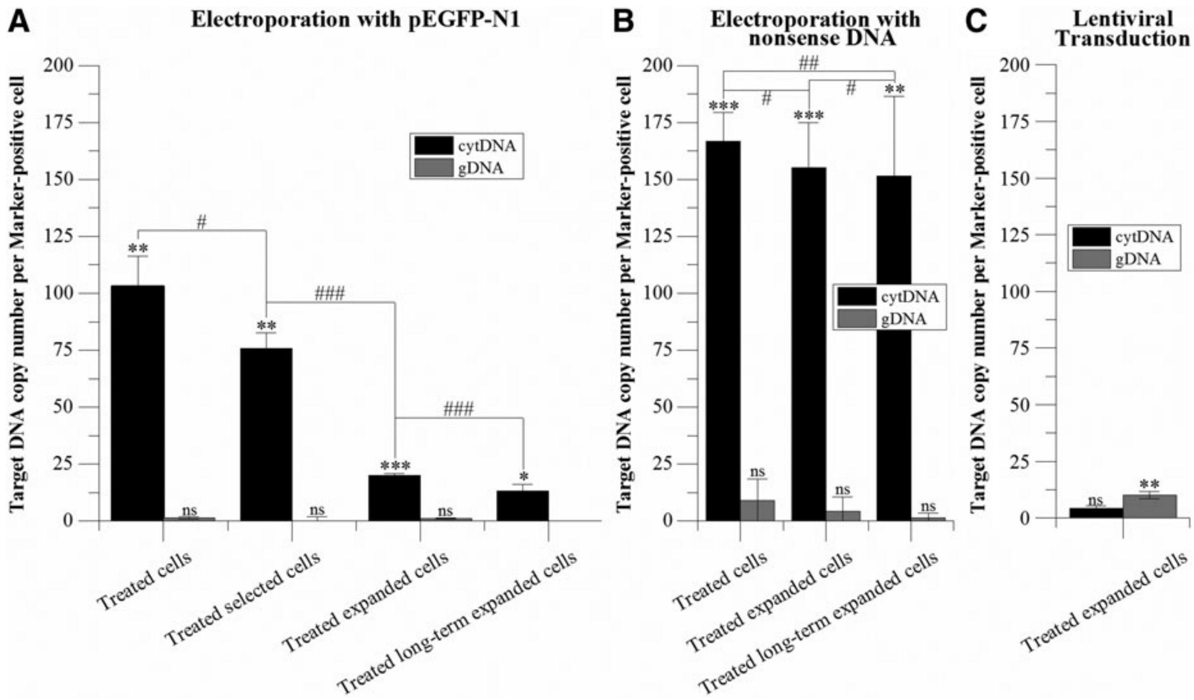

SUPPLEMENTARY FIG. S2. Target DNA copy numbers in target DNA-positive cells after electroporation or lentiviral transduction per marker-positive cell. qRT-PCR was performed using SYBR green and results were compared with a target DNA calibration curve (Supplementary Fig. S1). **(A)** Electroporation with pEGFP-N1, **(B)** electroporation with nonsense DNA, **(C)** lentiviral transduction of MSCs with a second-generation lentiviral vector system encoding for EGFP. ($n = 3$, mean ± SD; *Shows *t*-test comparison of each sample with the negative control: ***$p \leq 0.001$; **$p \leq 0.01$; *$p \leq 0.05$; ns, $p > 0.05$; $^{\#}$Shows *t*-test comparison between two samples; $^{\#}$shows *t*-test comparison of two samples: $^{\#}p \leq 0.05$; $^{\#\#}p \leq 0.01$; $^{\#\#\#}p \leq 0.001$). ns = not significantly different.

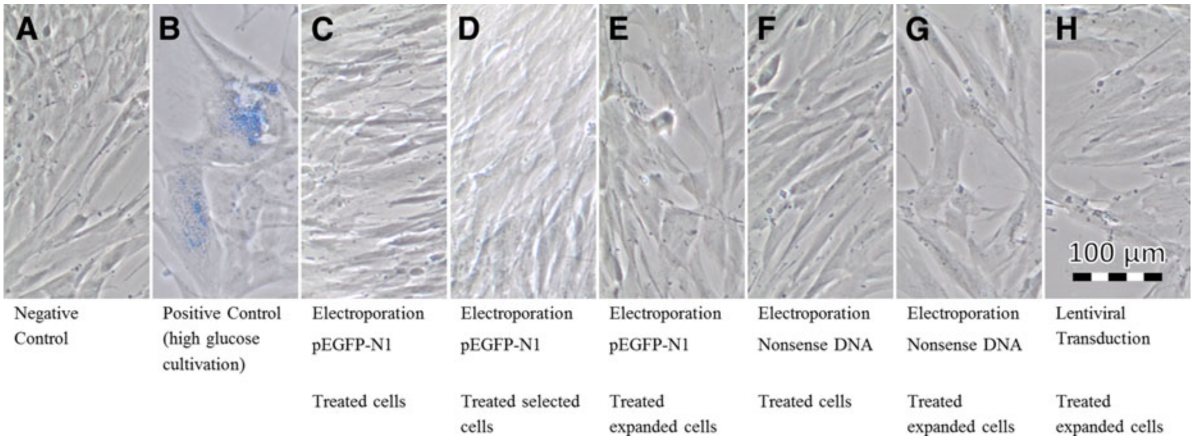

SUPPLEMENTARY FIG. S3. Examination of effects of electroporation and lentiviral transduction on senescence of AD-hMSCs. Representative images of senescence-associated β-galactosidase staining, which was performed after electroporation with pEGFP-N1 [treated cells **(C)**, treated selected cells **(D)**, and treated expanded cells **(E)**], electroporation with nonsense DNA [treated cell **(F)** and treated expanded cells **(G)**], and after lentiviral transduction [treated expanded cells **(H)**]. Nontreated cells were used as a negative control **(A)**, AD-hMSCs cultivated in high-glucose medium (4.5 g/L) for 21 days were used as positive control **(B)**. AD-hMSCs, human mesenchymal stem cells derived from adipose tissue; EGFP, enhanced green fluorescent protein.

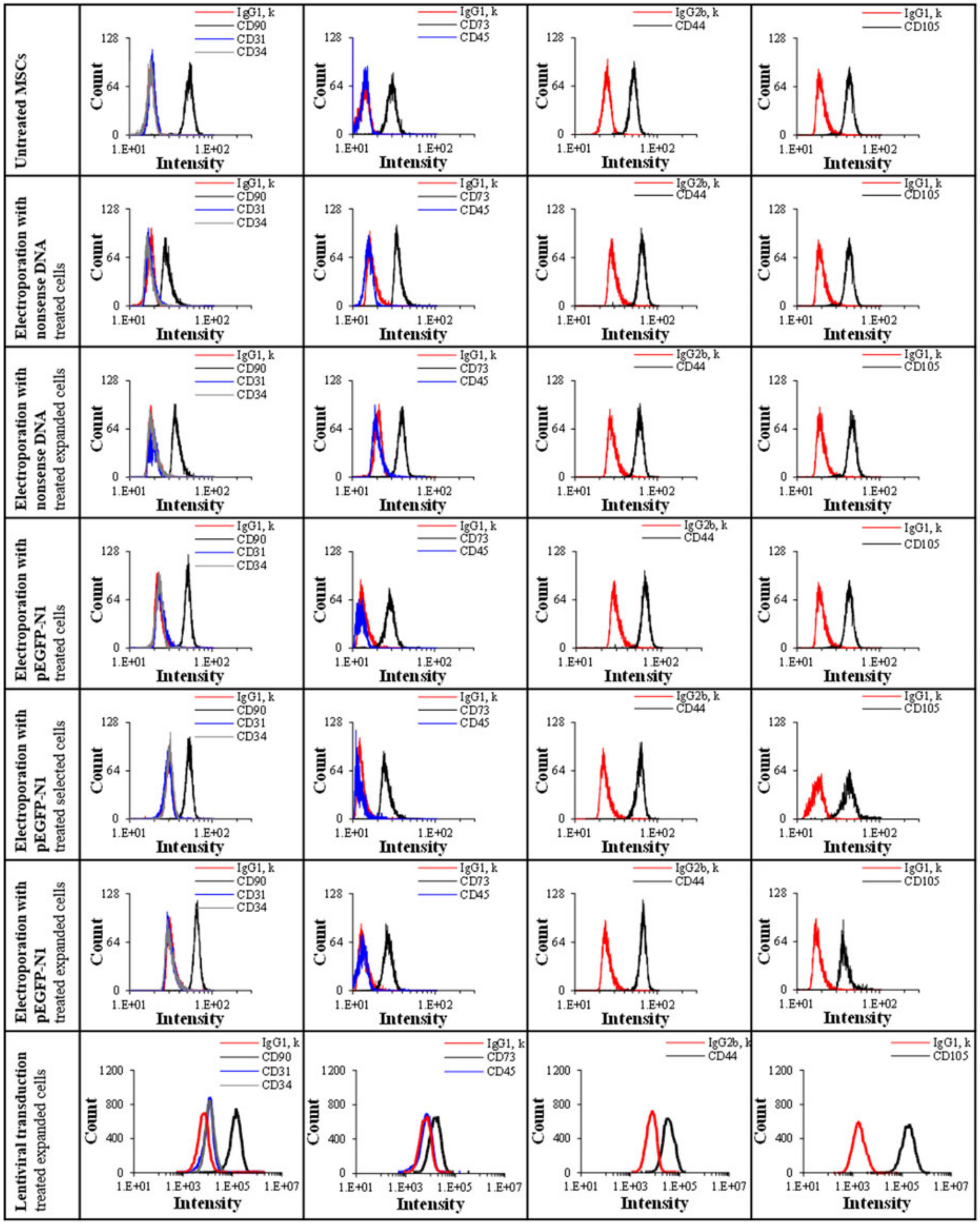

SUPPLEMENTARY FIG. S4. Flow cytometer analysis of surface genes of isolated AD-hMSCs in passage 1 (untreated cells) and after electroporation with pEGFP-N1-plasmid (EGFP-treated cells; EGFP-treated selected cells, and EGFP-treated expanded cells) or nonsense DNA (nonsense DNA-treated cells; nonsense DNA-treated expanded cells) or after lentiviral transduction (lentiviral transduction-treated expanded cells). Red lines indicate isotype controls, differently colored lines indicate specific antibodies.

Supplementary Table S1. Nonsense Label DNA Sequence

Forward	Reverse
C*C*G* C*T*T CAT GCT AAG GAT CTG GCT GCA TGC TAT GTT GAT ACG CCT AC/iCy3/ACT GCT CGA AGA AAA TAT ACG AAG CGG G*C*G* G*C*C	G*G*C* C*G*C CCG CTT CGT ATA TTT TCT TCG AGC AGT GTA GGC GTA TCA AC/iCy3/ATA GCA TGC AGC CAG ATC CTT AGC ATG A*A*G* C*G*G

*Phosphorothioate bonds; S instead of O.

Supplementary Table S2. Quantitative Real-Time Polymerase Chain Reaction Primer Sequences

	Forward	Reverse	Amplicon size
egfp	5′-CGA CCA CTA CCA GCA GAA C-3′	5′-CAG CAG GAC CAT GTG ATC G-3′	127 bp
Nonsense label-DNA	5′-CCG CTT CAT GCT AAG GAT CTG GCT GC-3′	5′-GGC CGC CCG TTC GTA TAT TTC TTC G-3′	80 bp
gap-dh	5′-AGG GCT GCT TTT AAC TCT GGT-3′	5′-CCC CAC TTG ATT TTG GAG GGA-3′	206 bp

SUPPLEMENTARY MOVIE S1. Time lapse recording of AD-hMSCs during selection. AD-hMSCs electroporated with pEGFP-N1 are shown during selection (cultivation in complete medium with 200 μg/mL G418 bisulfate). Images were taken every 2 min using the incubator microscope LumaScope 600 (Etaluma, Inc., USA) and a 40 $\times$ objective (LUCPlanFLN40X; Olympus, Japan); VirtualDub (Gnu Public License software by Avery Lee) was used to create videos at 72 fps. AD-hMSCs, human mesenchymal stem cells derived from adipose tissue; EGFP, enhanced green fluorescent protein.

SUPPLEMENTARY MOVIE S2. Time lapse recording of AD-hMSCs during expansion in complete medium. AD-hMSCs (electroporated with pEGFP-N1 and selected) are shown during expansion in complete medium. Images were taken every 2 min using the incubator microscope LumaScope 600 (Etaluma, Inc.) and a 40 $\times$ objective (LUCPlanFLN40X; Olympus); VirtualDub (Gnu Public License software by Avery Lee) was used to create videos at 72 fps.

SUPPLEMENTARY MOVIE S3. Time lapse recording of AD-hMSCs during osteogenic differentiation. AD-hMSCs (electroporated with pEGFP-N1, selected, and expanded) are shown during osteogenic differentiation. Images were taken every 2 min using the incubator microscope LumaScope 600 (Etaluma, Inc.) and a 40 $\times$ objective (LUCPlanFLN40X; Olympus); VirtualDub (Gnu Public License software by Avery Lee) was used to create videos at 72 fps.
