# Supplementary material for: Electroporation: A Sustainable and Cell Biology Preserving Cell Labeling Method for Adipogenous Mesenchymal Stem Cells
Source: Biores Open Access. 2019 Mar 29;8(1):32–44. doi: 10.1089/biores.2019.0001 (PMC6445215; doi:10.1089/biores.2019.0001)
Supplement: Supplemental data [file Supp_Table2.pdf]

Supplementary Table S2. Quantitative Real-Time Polymerase Chain Reaction Primer Sequences

	Forward	Reverse	Amplicon size
egfp	5′-CGA CCA CTA CCA GCA GAA C-3′	5′-CAG CAG GAC CAT GTG ATC G-3′	127 bp
Nonsense label-DNA	5′-CCG CTT CAT GCT AAG GAT CTG GCT GC-3′	5′-GGC CGC CCG TTC GTA TAT TTC TTC G-3′	80 bp
gap-dh	5′-AGG GCT GCT TTT AAC TCT GGT-3′	5′-CCC CAC TTG ATT TTG GAG GGA-3′	206 bp
