# Supplementary material for: Electroporation: A Sustainable and Cell Biology Preserving Cell Labeling Method for Adipogenous Mesenchymal Stem Cells
Source: Biores Open Access. 2019 Mar 29;8(1):32–44. doi: 10.1089/biores.2019.0001 (PMC6445215; doi:10.1089/biores.2019.0001)
Supplement: Supplemental data [file Supp_Fig1.pdf]

Supplementary Data

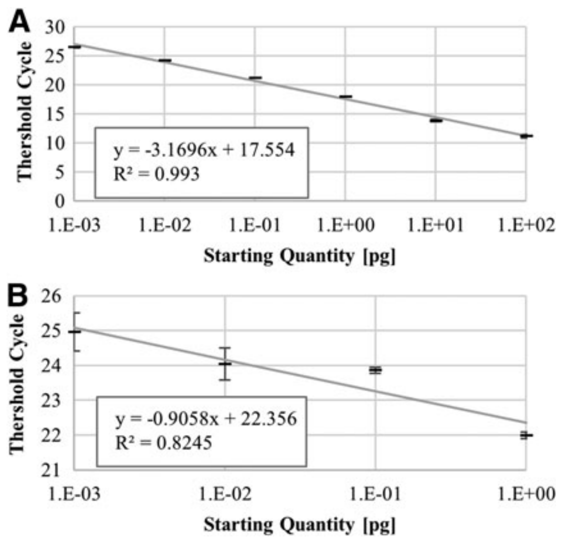

SUPPLEMENTARY FIG. S1. Calibration curves of qRT-PCR analysis. Samples of different known starting quantities of pEGFP-N1 **(A)** or nonsense DNA **(B)** were analyzed by qRT-PCR to obtain calibration curves. Data were exported using BioRadIQ5™ (BioRad, Germany). qRT-PCR, quantitative real-time polymerase chain reaction.
