# Supplementary material for: Electroporation: A Sustainable and Cell Biology Preserving Cell Labeling Method for Adipogenous Mesenchymal Stem Cells
Source: Biores Open Access. 2019 Mar 29;8(1):32–44. doi: 10.1089/biores.2019.0001 (PMC6445215; doi:10.1089/biores.2019.0001)
Supplement: Supplemental data [file Supp_Fig2.pdf]

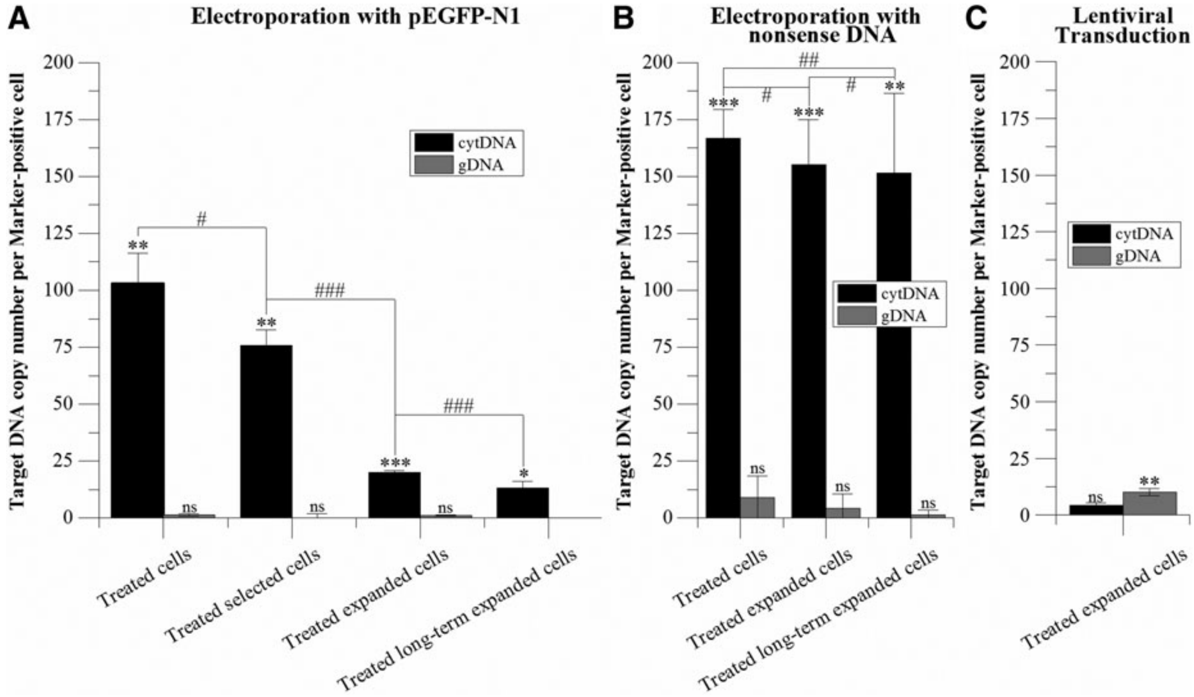

SUPPLEMENTARY FIG. S2. Target DNA copy numbers in target DNA-positive cells after electroporation or lentiviral transduction per marker-positive cell. qRT-PCR was performed using SYBR green and results were compared with a target DNA calibration curve (Supplementary Fig. S1). **(A)** Electroporation with pEGFP-N1, **(B)** electroporation with nonsense DNA, **(C)** lentiviral transduction of MSCs with a second-generation lentiviral vector system encoding for EGFP. ($n = 3$, mean ± SD; *Shows *t*-test comparison of each sample with the negative control: ***$p \leq 0.001$; **$p \leq 0.01$; *$p \leq 0.05$; ns, $p > 0.05$; $^{\#}$Shows *t*-test comparison between two samples; $^{\#}$shows *t*-test comparison of two samples: $^{\#}p \leq 0.05$; $^{\#\#}p \leq 0.01$; $^{\#\#\#}p \leq 0.001$). ns = not significantly different.
