# Supplementary material for: Electroporation: A Sustainable and Cell Biology Preserving Cell Labeling Method for Adipogenous Mesenchymal Stem Cells
Source: Biores Open Access. 2019 Mar 29;8(1):32–44. doi: 10.1089/biores.2019.0001 (PMC6445215; doi:10.1089/biores.2019.0001)
Supplement: Supplemental data [file Supp_Fig3.pdf]

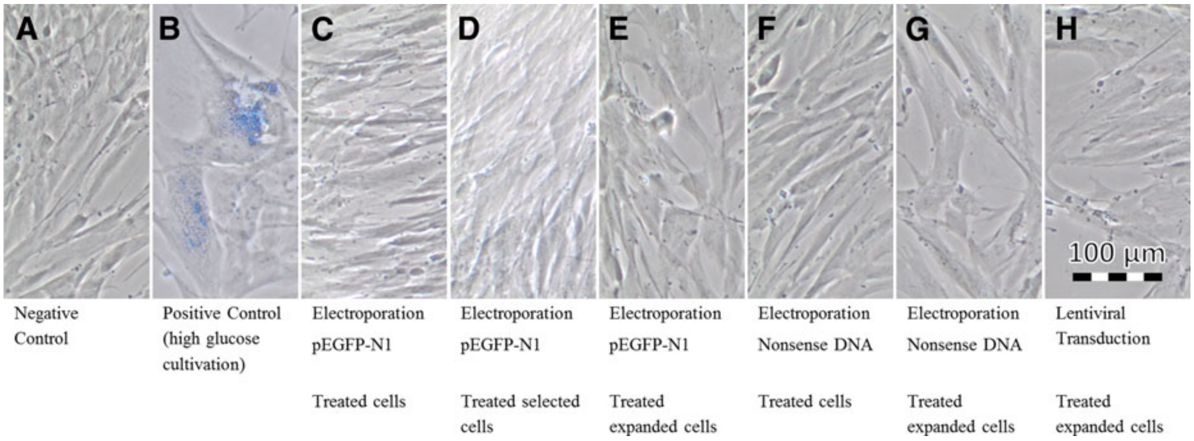

SUPPLEMENTARY FIG. S3. Examination of effects of electroporation and lentiviral transduction on senescence of AD-hMSCs. Representative images of senescence-associated β-galactosidase staining, which was performed after electroporation with pEGFP-N1 [treated cells **(C)**, treated selected cells **(D)**, and treated expanded cells **(E)**], electroporation with nonsense DNA [treated cell **(F)** and treated expanded cells **(G)**], and after lentiviral transduction [treated expanded cells **(H)**]. Nontreated cells were used as a negative control **(A)**, AD-hMSCs cultivated in high-glucose medium (4.5 g/L) for 21 days were used as positive control **(B)**. AD-hMSCs, human mesenchymal stem cells derived from adipose tissue; EGFP, enhanced green fluorescent protein.
