# Supplementary material for: Electroporation: A Sustainable and Cell Biology Preserving Cell Labeling Method for Adipogenous Mesenchymal Stem Cells
Source: Biores Open Access. 2019 Mar 29;8(1):32–44. doi: 10.1089/biores.2019.0001 (PMC6445215; doi:10.1089/biores.2019.0001)
Supplement: Supplemental data [file Supp_Fig4.pdf]

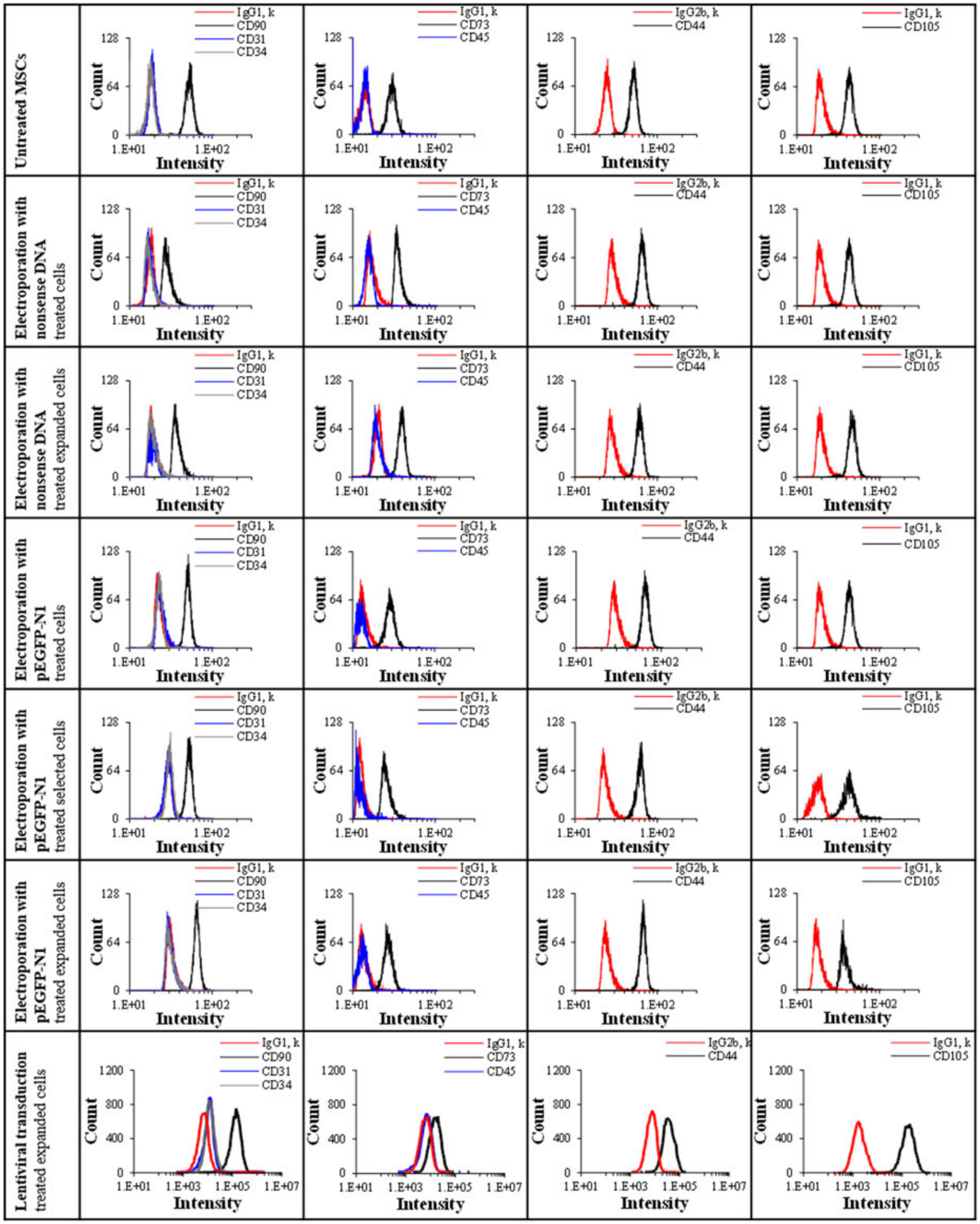

SUPPLEMENTARY FIG. S4. Flow cytometer analysis of surface genes of isolated AD-hMSCs in passage 1 (untreated cells) and after electroporation with pEGFP-N1-plasmid (EGFP-treated cells; EGFP-treated selected cells, and EGFP-treated expanded cells) or nonsense DNA (nonsense DNA-treated cells; nonsense DNA-treated expanded cells) or after lentiviral transduction (lentiviral transduction-treated expanded cells). Red lines indicate isotype controls, differently colored lines indicate specific antibodies.
